# Nutritionally Enhanced Food Crops; Progress and Perspectives

**DOI:** 10.3390/ijms16023895

**Published:** 2015-02-11

**Authors:** Kathleen L. Hefferon

**Affiliations:** Cell and Systems Biology, University of Toronto, Toronto, ON, Canada M5S 1A1; E-Mail: kathleen.hefferon@utoronto.ca; Tel./Fax: 1-(416)-978-5594

**Keywords:** biofortified crops, functional food, agricultural biotechnology, global health

## Abstract

Great progress has been made over the past decade with respect to the application of biotechnology to generate nutritionally improved food crops. Biofortified staple crops such as rice, maize and wheat harboring essential micronutrients to benefit the world’s poor are under development as well as new varieties of crops which have the ability to combat chronic disease. This review discusses the improvement of the nutritional status of crops to make a positive impact on global human health. Several examples of nutritionally enhanced crops which have been developed using biotechnological approaches will be discussed. These range from biofortified crops to crops with novel abilities to fight disease. The review concludes with a discussion of hurdles faced with respect to public perception, as well as directions of future research and development for nutritionally enhanced food crops.

## 1. Introduction

Plants grown as food crops possess a wide diversity of biologically active compounds which contribute to overall human health. The accessibility of food crops that are high in nutritional content is granted for those who live in the industrialized world; however, this is not always the case for the rural poor who reside in developing countries. For such populations, a diet that is balanced in adequate levels of vitamins and minerals can be difficult to achieve and maintain. All too often, a monotonous diet in which a single crop such as rice predominates is all that is on hand and affordable. Fortunately, due to recent developments in agricultural biotechnology, it is now possible to generate food crops which are nutritionally enhanced to improve the content and bioavailability of essential nutrients, such as iron and vitamin A [1,2,3,4]. Similar technologies have been used to fend off chronic illnesses including heart disease and cancer [5,6]. The following review describes some of the recent advances in agricultural biotechnology which have been undertaken to improve global health. 

## 2. Biofortification of Crops

### 2.1. Nutritionally Enhanced Food Crops to Improve Global Health

Today, approximately 842 million people around the globe are undernourished, meaning that they do not get enough food to eat [7]. Moreover, close to 2 billion of the world’s population suffers from “hidden hunger,” malnutrition caused not by too few calories, but by an inadequate intake of essential micronutrients in their daily diet [8]. People who suffer from malnutrition often consume meals which center around a staple crop and as a result lack access to the wide variety of fruits and vegetables that are required for a healthy diet. As a consequence, close to one -third of childhood deaths under the age of five worldwide stem from undernutrition, and one child in four is stunted due to inadequate nutrition [9]. Over the next few decades, as the world population approaches ten billion, and with the advent of climate change, achieving food security will pose an even greater challenge. The vast majority of global population increase will most likely take place in the developing world, and global warming is expected to result in drought, flooding, and severe temperatures. The development of plants which are nutritionally enhanced and resistant to abiotic stresses presents a viable solution to these future challenges [10,11].

Traditionally, vitamins and minerals have been added to food crops through supplementation or biofortification practices. The provision of micronutrients in the form of supplements to malnourished populations has proven to be successful [12,13]. However, the extent of this success is unclear and such strategies are prone to fail in areas of scattered small populations or regions that become politically unstable. The use of supplementation programs may still fall short of the goals set in place by international health organizations. Besides the expense, there is often too high a level of noncompliance among the population group that the supplementation program endeavors to help [1,4].

Biofortification of crops can take place either by adding the appropriate mineral or inorganic compound to fertilizer, by conventional plant breeding, or through the use of biotechnology. Although the application of fertilizers biofortified with micronutrients is the most simple of these methods, this practice can be confounded by the differences in mineral mobility and accumulation among plant species and different soil compositions in the specific geographical location of each crop, making success of this method highly variable. It is also necessary to apply the micronutrient regularly to the soil, therefore increasing both cost and labor.

The particular species of micronutrient ingested is also important. For example, the organic species of a particular micronutrient can be more easily incorporated into tissue proteins such as red blood cells and skeletal muscle [14]. Organic species of micronutrients can also be stored more effectively by the body and micronutrient status retained for longer periods of time than inorganic micronutrients [15].

Another drawback is that it is not always possible to target the micronutrient into edible plant tissues and so this technique is only successful using certain plant species and mineral combinations. Biofortification of food crops with minerals such as selenium, iodine and zinc have been achieved using this strategy [16,17,18,19]. The design of conventional plant breeding programs to improve micronutrient content has also proven to be successful; however, there are limitations with respect to the amount of variability in the plant gene pool and the time needed to generate cultivars with the desired trait(s) [20,21,22,23,24,25].

As an alternative, the generation of micronutrient-dense biofortified crops through the use of biotechnology is at once more cost-effective, sustainable and realistic. With transgenic crop technology, the genes of interest are inserted directly into the plant genome and the resulting recombinant proteins which are expressed may not be feasible under conventional plant breeding programs. Conventional breeding that can acquire and retain specific traits while not compromising others can be complex and comes with its own challenges. In many occasions, it would be impossible to breed for a specific trait using conventional means, and the timescale and effort involved may be quite unrealistic. While a certain amount of time and effort is initially involved in generating transgenic plants, the germplasm can be maintained at a low cost, in a timely manner, and without the need for nutrition-based organizational programs. Expected benefits of consumer traits has been estimated for some genetically modified (GM) crops as compared to their conventional counterparts [26,27,28].

Recently, a new line of biotechnology based on the principle of genome editing has come to the forefront. Genome editing focuses on nuclease-based forms of engineering such as the TALENS (transcription activator like effector nucleases) or the CRISPR (clustered regularly interspaced short palindromic repeats)/CRISPR-associated (Cas) systems, and concerns the creation of precise incisions, mutations and substitutions in plant and other eukaryotic cells [29,30]. This technology will revolutionize the way we think about enhancing food crops to improve global nutritional status. It is less likely that new varieties of crops which harbor the small nucleotide modifications that are created by genome editing will be subjected to the same strict set of regulations as are currently held for transgenic crops. As a result, genome editing may very well help plant biotechnologists avoid the same public controversy surrounding GMOs [31]. Genome editing has been performed on crops such as barley, rice, tobacco, maize and arabidopsis, and is currently in a preliminary stage of development [32,33,34,35,36,37,38].

The generation of biotech food crops with improved attributes, such as increases in iron storage protein or greater levels of folate can provide sufficient levels of these and other much needed micronutrients that are frequently lacking in the diets of developing world [39,40]. Not only must these micronutrients be generated at high levels in plants, they must also be readily bioavailable, or absorbed and utilized by the body so that a consumer’s micronutrient status is improved even upon cooking or processing the food in the manner that a particular culture practices. It is just as important that the biofortified crop be accepted by the community it is generated for and that it is readily adapted by farmers in significant enough numbers to improve a given community’s general nutritional health [41,42]. This can at times be problematic as some given populations remain wary of the use of genetically modified foods. The following section provides examples of biofortified food crops using biotechnology that are under development. 

#### 2.1.1. Biofortified Rice

Each year, vitamin A deficiency causes eye damage in three million preschool-aged children. Of these; half a million become blind and two-thirds will die shortly afterwards. The precursor molecule required for vitamin A biosynthesis, β-carotene, is absent from the grain of cereals such as rice. As a result, many with a largely monotonous diet are at risk of vitamin A deficiency. Golden Rice, named for its golden colour due to its high β-carotene content and generated using biotechnology, was designed by the research group of Ingo Potyrus and offers a viable solution. This transgenic crop was engineered with two genes from other organisms (daffodil and the bacterium *Erwinia uredovoia*) which reconstitute the carotenoid biosynthetic pathway within the rice genome [43]. This new trait was then transferred into high yielding local commercial cultivars by marker-assisted back-cross breeding. The most current Golden Rice technology, known as GR2, utilizes genes from two distinct provitamin A pathways, including the substitution of the phytoene synthesis gene from maize for the analogous daffodil gene used in GR1 rice [44,45]. Golden rice can produce levels of β-carotene that were up to 35 µg/g of dry rice [46]. Servings of 130–200 g of deuterium-labeled Golden rice grown hydroponically in heavy water expressing 0.99–1.53 mg β-carotene were fed to human volunteers. Blood samples taken at 36 days exhibited 0.34–0.94 µg retinol, indicating that β-carotene derived from Golden rice is effectively converted to vitamin A at a rate of 500–800 µg retinol per 100 g uncooked Golden rice, close to the recommended daily allowance for children [46]. The vitamin A value of Golden rice, nontransformed spinach and β-carotene provided in oil to children were also compared, and the results of this study showed that the β carotene derived from Golden rice was just as effective as pure β-carotene and in fact more effective than β-carotene provided from spinach in providing vitamin A to children [47]. Together, these results suggest that Golden rice could realistically be used to alleviate Vitamin A deficiency in rice-consuming populations [48]. Golden Rice could be considered the very first genetically engineered crop that was specifically designed to combat malnutrition. The select advantage of a biofortified crop such as Golden Rice is that it could readily reach remote rural populations which have no access to supplementation programs [49,50,51,52].

Rice has also been engineered to combat other major forms of malnutrition, including iron and folate deficiency. These were addressed by improving iron storage and transport proteins in plants and by adding a phytase to improve ion absorption in the gut [53,54,55,56,57]. Transgenic rice which expresses essential amino acids such as free lysine have also been developed using RNAi silencing-based technologies. De Steur *et al.* (2012) [58] demonstrated that transgenic biofortified rice could be cost-effective in alleviating folate deficiency rather than conventional supplementation programs. Iron has been increased in rice as a result of conventional plant breeding rather than the development of transgenic plants. Haas *et al.*, 2005 [59], demonstrated that Filipino women consumed 1.79 mg iron per day in the form of steamed biofortified conventional rice. A control group consumed nonbiofortified rice at a level of 0.37 mg of iron/day. Studies using transgenic rice that have been biofortified with iron have centered around the overexpression of iron storage proteins such as ferritin. Rice cultivated from these transgenic plants contain 3–4 times as much iron as their wild-type counterparts [60,61].

Phytic acid (PA) is a known inhibitor of zinc absorption and is prevalent in many cereals. Phytic acid binds to zinc and other minerals such as iron to form an insoluble complex in the gastrointestinal tract that prevents mineral absorption. Since the prevalence of phytic acid in cereals such as wheat, corn and rice can have serious nutritional consequences efforts have been made to reduce its content [62,63,64]. Standard methods used for the reduction of phytic acid include heat or microwave treatment, as well as the use of chemicals such as hydrochloric acid or acetic acid. The exogenous application of recombinant microbial phytase is also frequently used to reduce the level of phytic acid in grain [65]. Since phytic acid can be effectively degraded by microbial phytase enzymes, resulting in an increase in mineral availability, transgenic corn expressing phytase derived from *Aspergillus niger* has been generated. These transgenic varieties were found to be as effective at lowering phytic acid levels as conventional corn that was supplemented with commercially used phytases [66]. Recently, cereal mutants exhibiting a low phytic acid (lpa) phenotype have also been developed in rice, wheat and maize [67]. Although effective, these strategies are sometimes associated with downstream impacts on crop yield and other parameters concerning agronomic performance. To meet this challenge, transgenic rice crops have been developed by manipulating the phytic acid biosynthetic pathway through RNAi-mediated gene silencing of the *IPK1* gene, which is involved in catalyzing the final step of phytic acid biosynthesis [68]. Agronomic traits associated with these transgenic plants were also analyzed to ensure that the plants were not compromised in any physiological way. This strategy led to the generation of rice plants with greatly reduced yields of phytic acid and correspondingly improved mineral bioavailability.

#### 2.1.2. Biofortified Maize and Cassava

Maize has also been biofortified with β-carotene as well as other essential micronutrients necessary to maintain one’s health. Li *et al.* (2010) [69] measured the triglycerol-rich lipoprotein fraction of blood from North American female volunteers who consumed biofortified maize porridge. In this case, the authors found a vitamin A equivalence value of β-carotene in biofortified maize to be 3.1-fold higher than in conventional white porridge maize [69]. A similar study using Zimbabwean men found biofortified yellow maize porridge to provide an equivalence of 40%–50% of the US recommended Dietary Allowance of vitamin A [70]. Another study [70] using Mongolian gerbils who were fed biofortified maize containing β-cryptoxanthin resulted in a more efficient bioconversion than the use of a β-carotene supplement. The results of these studies indicate that the biofortification of maize via biotechnology can be a useful strategy to improve vitamin A status. A triple-vitamin fortified maize which expresses high amounts of β-carotene, ascorbate, and folate has been developed in the endosperm through metabolic engineering [69]. The transgenic kernels contained 169-fold the normal amount of β-carotene, 6-fold the normal amount of ascorbate, and double the normal amount of folate as conventionally-bred crops. Crops such as these can offer far more nutritionally complete meals for Africa’s malnourished [70].

The BioCassava Plus project has been developed to target cassava, the nutritionally deficient staple of a quarter of a billion sub-Saharan Africans. Cassava with high levels of β-carotene has been produced and fed to healthy volunteers in the form of a porridge [71]. Blood samples taken from these volunteers demonstrated that biofortified cassava increases β-carotene and retinyl palmitate TRL plasma concentrations [71]. The results of this study suggest that biofortified cassava could be used to prevent vitamin A deficiency. Programs such as these could therefore generate cassava crops with more lasting nutritional benefits [72]. Cassava roots also express a low protein: energy ratio, and less than 10%–20% of the required amounts of iron, zinc, vitamin A and vitamin E. By reducing levels of the toxin cyanogen in roots, iron root uptake and protein accumulation in cassava could be enhanced [73]. Crops biofortified with multiple micronutrients have also been generated [74,75].

#### 2.1.3. Biofortified Wheat

Wheat has been altered using biotechnology for a number of health benefits. For example, levels of celiac-disease causing gliadins have been lowered from wheat using RNAi-based technologies, and the level of free lysine, an essential amino acid that is generally scarce in wheat, has been increased. Genetically altered wheat has been tested for dough making quality and taste with encouraging results. Biofortified wheat provides more options for the proportion of the population who are gluten sensitive or intolerant, and can also provide higher levels of micronutrients, such as iron and zinc, to those in developing countries who use wheat as a staple [76,77,78]. 

Wheat has also been under study as a model crop for zinc biofotification. Zinc (Zn) deficiency ranks as the fifth leading cause of disease in low-income countries, and affects billions of people whose diet is based on cereal grains low in Zn content. Health defects due to zinc deficiency include stunted growth, poor immunity, impairments in mental development and birth complications. Recently, improvements in nitrogen (N) management has enabled Zn concentrations in grain crops such as wheat to be improved, both through Zn available in soil as well as by foliar application. Radiolabelled ^65^Zn has been shown to be taken up by the roots, translocated to shoots and to accumulate in the wheat grain [79,80]. Erenoglu *et al.*, 2011 [81], demonstrated that increasing the nitrogen supply in the soil can stimulate the root-to-shoot translocation of Zn and enhance its accumulation in wheat grain, possibly via increasing the abundance of transporter proteins in the presence of nitrogen [81]. Nitrogen availability therefore represents a key component for the zinc biofortification of wheat and thus can improve the nutritional status for many who reside in developing countries. 

Selenium, which has demonstrated chemoprotective properties, is another micronutrient that is found in deficient levels in soil, and as a result, is often only present in low quantities in grain crops [82,83,84]. Moreover, selenium present in its organic form, such as selenomethionine and selenocysteine, are significantly more bioavailable than inorganic Se species. Recently, Pobaciones *et al.*, 2014 [84], demonstrated that chickpea is capable of accumulating high concentrations of Se in the grain, by measuring the effect of different Se doses in the form of fertilizer on grain yield and the Se bioavailability in grain. The authors demonstrated that chickpea could accumulate sufficient amounts of Se-Met with only low amounts of Se-containing fertilizer applied as a foliar spray at the start of flowering.

#### 2.1.4. Nutritionally-Enhanced Feed Crops

Nutritionally enhanced crops have been designed to address improvements in feed for livestock and poultry. For example, animal feed crops have been generated which produce higher levels of limiting amino acids so that fewer supplements will be required. Feed crops have also been developed with the aim of producing more environmentally friendly manure. As an example, poultry and swine fed transgenic maize with increased free lysine content increased body weight gain to a level comparable with animals fed diets with lysine as a supplement. Similar results have been demonstrated for livestock fed soybean and lupin [85]. In the same way, Tong *et al.*, 2014 [86], cloned bacterial aspartate kinase and adenylylsulfate reductase genes into alfalfa plants, thus providing increases of sulfur containing amino acids cysteine and methionine levels by 30% and 60%, respectively [86]. An increase in the abundance of other essential amino acids such as aspartate and lysine was also observed. Strategies such as these can help to enhance the nutritional value of feed crops used for livestock. 

Transgenic maize crops expressing cell wall invertase have also been developed [87]. Grain yield was substantially improved (up to 145.3%) in transgenic as compared to wild-type maize due to enhanced grain size and number. Constitutive expression of invertase increased total starch content in the transgenic kernels as well, demonstrating that this gene can be utilized to improve both grain yield and grain quality in crop plants.

The removal of anti-nutrients from animal feed to increase micronutrient absorption has been explored using biotechnology. For example, transgenic maize producing a high level (779,800 U/kg) of the β-glucanase Bgl7AM was found to be stable at pH 1.0–8.0, the normal environment of the digestive tract [88]. These crop plants simplify the feed processing procedure, making the feed more amenable for livestock consumption.

### 2.2. Plants with Other Health Benefits

Not only can food crops be biofortified by genetic engineering, they can also be designed to contain bioreactive compounds which have improved health benefits or reduce the risk of chronic diseases, such as cancer and heart disease [89,90]. The following section provides two examples of these “designer crops”.

Plant seed storage oils have been examined for their ability to produce novel fatty acids that are beneficial to human health. For example, a variety of “designer oilseed” transgenic plants have been developed through metabolic engineering to synthesize omega-3 fatty acids found routinely in fish oils [91]. Omega-3 long-chain polyunsaturated fatty acids (omega-3-FA) are of great interest due to their dietary benefits such as improvements to brain function and development as well as for cardiovascular health. Since most omega-3 FA comes from marine life and the seas have been overfished, plants represent a more sustainable source of this nutrient. Genetically modified plants, algae and krill are under development to express levels of omega-3 FA approaching those found in marine organisms [92,93]. The metabolic pathway to produce this fatty acid has been reconstituted in plants such as false flax, a relative to canola, via metabolic engineering [94,95]. Other beneficial fatty acids have also been made in plant seed oils, including γ-linolenic and stearidonic acid, as well as arachidonic acid [96].

Expression in tomato plants of the antioxidant anthocyanin, a compound found in blueberries and cranberries, through metabolic engineering resulted in tomato fruit which could extend the life spans of cancer-susceptible mice by up to thirty percent [97]. These tomatoes, which are also believed to fight cardiovascular disease and exhibit anti-inflammatory properties, have been produced as a juice and are currently being tested on heart patients in Britain. Tomatoes were chosen because they are consumed by large amounts of the population and are quite affordable, unlike blueberries, which tend to be seasonal and higher priced. In the future, these tomatoes may make their way into food products such as ketchup and pizza sauce.

Tomatoes have also been transformed with the gene encoding grape (*Vitis vinifera* L.) stilbene synthase under the control of a fruit specific promoter. Transgenic tomato plants accumulated trans-resveratrol and trans-resveratrol-glucopyranoside, and the levels during tomato ripening reached up to 53 µg/g fresh weight [98]. Resveratrol is a bioactive compound found in grapes and red wine but not many other common food sources, and so producing resveratrol in tomatoes can improve their nutritional value. Significant increases in both antioxidant capability and ascorbate content were found in these tomato lines. Transgenic tomato fruit were able to counteract the pro-inflammatory effects of phorbol ester in a culture of monocyte-macrophages [99,100]. 

The metabolic pathway of isoflavones found in soybean has been expressed in transgenic tobacco lines. Leaves from these tobacco plants exhibited elevated isoflavone synthesis, and estrogen-deficient mice fed transgenic leaf extracts exhibited reduced osteoclast number and expression of osteoclastogenic genes, as well as higher total serum antioxidant levels and increased uterine estrogenicity compared with control mice [101]. 

## 3. Biopharmaceuticals Produced in Plants

The generation of biopharmaceuticals in plants adds another layer of complexity to the role of plants in human health. Vaccines and other therapeutic proteins including monoclonal antibodies can be produced in plants and now a handful are in the early stages of commercialization. Today, the variety of therapeutic proteins produced in plants is considerable, and ranges from human monoclonal antibodies against HIV to vaccine proteins against smallpox and other potential biological warfare threats, and even to an assortment of anti-cancer therapeutic agents for the newly emerging field of personalized medicine. Molecular farming as a field originally stemmed from the need for safe and inexpensive biopharmaceuticals in developing countries. These vaccines are easily transportable and do not require refrigeration to ensure that they are accessible to remote regions of the planet. Vaccines produced in food crops including soybean, tomato and banana can be directly consumed and effectively elicit an immune response to a particular pathogen. Plants expressing vaccine proteins can be raised using local farming techniques and need only be partially processed, features that can substantially reduce production costs. Bananas expressing biopharmaceuticals can be dried into chips, or tomatoes can be lyophyllized into a powder and reconstituted as a juice when needed [102].

The earliest research and development on plant made pharmaceuticals focused upon diarrheal infectious diseases which are major causative agents of infant mortality in developing countries, such as cholera, rotavirus and Norwalk virus [69]. Today, clinical trials are underway to examine the ability of plant-derived therapeutic proteins to treat challenging diseases such as HIV and Ebola virus [103]. Some of these therapeutic proteins are generated in transgenic plants, however, an increasing number are generated from plant virus expression vector systems [104]. Plant viruses have been engineered in the form of modules and can produce very large amounts of the protein of interest within a short period of time (most often days). Transgenic plants remain preferable in certain cases as they can generate transgenic seed, something that cannot be realized with a transient expression system based on a plant virus. Other production systems which center on chloroplasts and hairy roots also exist [105,106].

### 3.1. Public Perception and Politicization of Nutritionally Enhanced Crops

While crops which are nutritionally enhanced through biotechnology could clearly play a role in alleviating malnutrition for developing countries, a number of issues must still be addressed [107]. Among the most daunting is public perception and politicization, and how these factors will influence the regulation of biotech crops in the future.

Today, the viewpoints between those who advocate for agricultural biotechnology and those who oppose it could not be more polar [108]. Opposition to GM crops revolve around issues such as biosafety or the vulnerability of farmers and food to corporate monopolies. Non-governmental organizations such as Greenpeace go out of their way to motivate opposition for genetically modified organisms (GMOs). These groups act by diffusing information and heightening anxiety with the mass public as well as with public authorities. Such groups deliberately operate at a sufficient distance from their scientific counterparts so that their claims tend to be left unchallenged by those familiar with the “sound science” involved with this technology. False stories range from Indian livestock dying from consuming the leaves of Bt cotton crops to Indian farmers committing mass suicide as a result of the assault of GMO crops on their livelihood [109,110]. Most recently, a highly discredited study claiming that GM foods increased the number of tumors in mice has induced a sort of mass hysteria among opponents of GMOs [110]. Although the paper later was retracted, it was highly cited by opposition groups and added fuel to the fire of the GMO controversy.

Public anxiety regarding misinformation over GMOs in foods has reached a new level of hysteria such that food companies with financial interest in opposing them have spent billions of dollars advertising their foods as “GM-free”. This reinforces anti-biotechnology sentiments and upholds the mistaken belief that harm will come to those who consume foods made up of GM ingredients. Most recently, a representative of the grocery store Whole Foods is claiming that by 2018, any products they sell that contain GMOs will have a cigarette-like label and warning [111]. The stigma regarding GMOs has reached epidemic levels with the push for US state legislatures to pass bills for mandatory labeling of GMO products [112]. In the past year alone, Proposition 37, the GMO labeling mandate for California has failed; however, the battle with this and other states is far from over.

In order to determine their safety on the environment and human health, all GM crops must undergo risk assessment which is both rigorous and thorough. These regulations revolve around a detailed analysis regarding the molecular characterization of the crop, an assessment of the crop’s potential for toxicity and/or allergenicity, and a nutritional analysis of the crop. In Europe, this process has become hindered by political opposition, and GM crops with all kinds of potential to help the world’s most needy are currently bogged down in bureaucracy. The structure of the European regulatory program has brought about a virtual moratorium of GM crops across the EU, thus blocking research and development of nutritionally enhanced crops from moving forward for humanitarian aid [113]. The best example of this moratorium in action is that of β-carotene biofortified Golden Rice, which has still not been approved for release in spite of its urgent need and readiness for well over a decade [114,115,116,117,118,119].

### 3.2. Commercial and Approval Status of Nutritionally Enhanced Crops

In 2013, the 18th year of commercialization of biotech foods, a record 175.2 million hectares of biotech crops were grown globally up from 170 million hectares in 2012. The fact that the proportion of GM crops that are planted and replanted from year to year is virtually 100% provides a strong indication that farmers are pleased with the performance of this new technology. Even more interesting is the fact that over 90%, or >16.5 million of those who planted GM crops, were small, poor farmers in developing countries [120]. This contradicts previous views that biotech crops would only be adapted by industrialized countries.

GM crops with two or more stacked traits, such as herbicide tolerance and insect resistance, were grown in 13 different countries in 2013, ten of which were developing countries. GM crops with stacked traits represented 27% of the total amount of land used for GM crops globally, and the number of biotech plants with two or more transgenic traits is growing. Stacked traits can be generated either by cross-breeding of two GM plants or by the retransformation of a GM plant with an additional transformation cassette. Since it is entirely possible that parental plants may be significantly heterozygous, the resulting offspring may display high phenotypic variability and will make comparisons with non GM plants, for example, more complex. This must be taken into account when regulatory bodies perform risk assessments [121].

According to current regulatory practice within the EU, stacked events are considered to be new GMOs and are treated as such. Prior to marketing they require the same regulatory approval, including an assessment of their safety, as is needed for single transformation events [122]. In the EU, GM maize and oilseed rape stacked events have already been evaluated with respect to their risks for the environment and for human or animal health [90]. Other countries such as the United States may not require this depth of regulation.

At least 36 countries have been granted regulatory approval for GM crops since 1994, with Japan having the highest number of GM events approved, followed closely by the USA. Three countries in Africa (South Africa, Burkina Faso and Sudan) are currently commercializing GM cotton and maize with great success, and seven others are undergoing field trials and taking steps toward commercialization. New GM crops that would be particularly helpful for poor African farmers, such as insect resistant cowpea, are also under research and development [123].

While the vast majority of crops which have been approved for commercialization focus on pest resistance and herbicide tolerance, a number of nutritionally enhanced food crops have also undergone safety approval [124]. In the industrialized world, plants with improved fatty acid content, such as omega-3-FA are now available, and the cancer fighting tomatoes discussed earlier may reach approval status in the US within the next two years [97].

## 4. Conclusions

In spite of opposition groups, GM crops now account for more than 300 million acres worldwide and are grown by over 17 million farmers in more than 25 countries. The vast majority of the increase in farming of GM crops is in developing countries. In 2012, the World Health Assembly (WHA) agreed on a set of global targets to hold the world accountable for reducing malnutrition [125]. It is unlikely that these targets will be met within the timeframe set and new Sustainable Development goals are now being set up with the target date of 2030. To achieve the goal of providing crops with additional health benefits on a global scale, much work is required and will involve interactions between many disciplines including plant breeders, molecular biologists, nutritionists and even social scientists. It is not worthwhile to spend the effort generating a biofortified crop for a given population if they are knowledgeable, prepared and not already willing to accept the technology or any changes in appearance of the biofortified crop [126,127,128,129,130]. New crop varieties with enhanced nutritional qualities must be evaluated by clinical trials, and select populations who can benefit most from them must be educated so that they understand how these advantages can make a difference in their community’s overall health [4,131,132,133,134,135,136,137,138,139,140,141,142,143]. Research and development of nutritionally enhanced “orphan crops” sorghum, millet, and pigeon pea, which are important to the world’s poor but overlooked by industrialized countries, must also be implemented. Cooperative efforts between governments, industry and nonprofit organizations will truly eliminate hunger from the world’s rural poor.
